# Optimizing Oxygen-Production Kinetics of Manganese Dioxide Nanoparticles Improves Hypoxia Reversal and Survival in Mice with Bone Metastases

**DOI:** 10.1021/acs.molpharmaceut.3c00671

**Published:** 2024-02-16

**Authors:** David A. Murphy, Daniela Osteicochea, Aidan Atkins, Caitlin Sannes, Zachary McClarnon, Isaac M. Adjei

**Affiliations:** Department of Biomedical Engineering, Texas A&M University, College Station, Texas 77843, United States

**Keywords:** bone metastases, tumor microenvironment, hypoxia, nanoparticle, natural killer cells, immune suppression

## Abstract

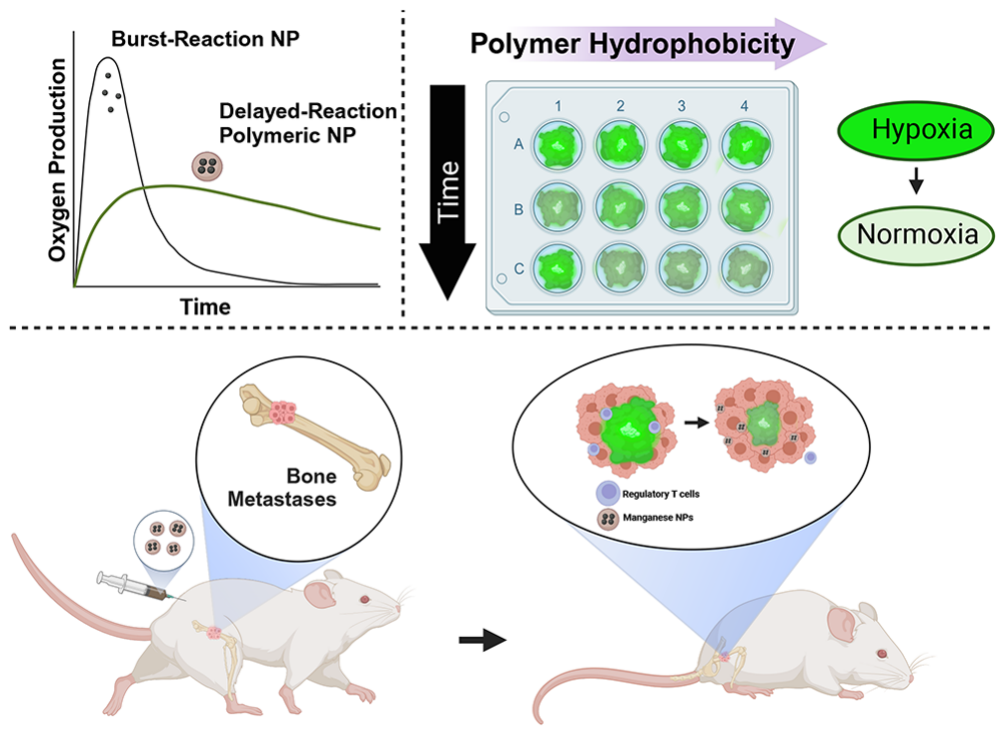

Persistent hypoxia in bone metastases induces an immunosuppressive environment, limiting the effectiveness of immunotherapies. To address chronic hypoxia, we have developed manganese dioxide (MnO_2_) nanoparticles with tunable oxygen production kinetics for sustained oxygenation in bone metastases lesions. Using polyethylene glycol (PEG)-stabilized MnO_2_ or poly(lactic[50]-*co*-glycolic[50] acid) (50:50 PLGA), poly(lactic[75]-*co*-glycolic[25] acid) (75:25 PLGA), and polylactic acid (PLA)-encapsulated MnO_2_ NPs, we demonstrate that polymer hydrophobicity attenuates burst oxygen production and enables tunable oxygen production kinetics. The PEG-MnO_2_ NPs resulted in rapid hypoxia reduction in spheroids, which was rapidly attenuated, while the PLA-MnO_2_ NPs exhibited delayed hypoxia control in cancer spheroids. The 50:50 PLGA-MnO_2_ NPs exhibited the best short- and long-term control of hypoxia in cancer spheroids, resulting in sustained regulation of the expression of HIF-1α and immunosuppressive genes. The sustained control of hypoxia by the 50:50 PLGA-MnO_2_ NPs enhanced the cytotoxicity of natural killer cells against cancer spheroids. In vivo, 50:50 PLGA-MnO_2_ showed greater accumulation in the long bones and pelvis, common sites for bone metastases. The NPs decreased hypoxia in bone metastases and decreased regulatory T cell levels, resulting in enhanced survival of mice with established bone metastases.

## Introduction

Recent advances in cancer therapies have improved the survival rates for patients diagnosed with early-stage disease.^1,2^ However, bone metastases negatively impact patient survival, with treatment limited to palliative therapies to decrease pain and fractures.^3,4^ The impact of bone metastases on survival is significant, as it negatively impacts the efficacy of immunotherapies, particularly checkpoint inhibitors. The bone marrow environment and bone metastases are immunosuppressive, inhibiting the infiltration and function of cytotoxic immune cells necessary for immunotherapies to function.^3,5^

The bone marrow is hypoxic to maintain the hematopoietic stem cell niche.^6−8^ Hypoxia limits the antitumor function of immune cells by upregulating anti-inflammatory ligands such as PD-L1, resulting in an immunosuppressive environment capable of limiting patients’ responses to growing tumors.^9−14^ The TME also alters MHC class 1 expression, allowing tumor cells to avoid cytotoxic T cell recognition.^10,13−16^ T cell metabolism is adversely affected by decreasing hypoxia levels, diminishing their activity.^17,18^ Regulatory T cells also increase in population in hypoxic environments.^19,20^ Hypoxia upregulates CD73 expression, increasing the generation of adenosine from extracellular ATP.^21,22^ The increased adenosine produces anti-inflammatory effects via A2A receptor activation and increases the population of tumor-associated macrophages (TAM), which act to attenuate immune activity.^17,21,23−26^ Hypoxic stress also produces reactive oxygen species (ROS), such as hydrogen peroxide (H_2_O_2_), which can act as an immunosuppressive modulator by promoting the efflux of ATP from the cell.^15,27,28^

Reducing hypoxia in the TME can improve the efficacy of immunotherapies by restoring the antitumor activity of the immune system.^23,29^ Exposure to hyperoxic oxygen levels improved outcomes for mice with experimental systemic metastases via T-cell-mediated tumor rejection.^21,23^ However, the overexposure of oxygen in the lungs causes inflammation, oxygen toxicity, and other side effects, prompting a need to develop a system capable of sustained and localized release of oxygen.^15,30,31^ Nanocarriers are being explored in providing hypoxia relief to tumors. These nanoparticles carry reactive ingredients such as manganese dioxide, calcium peroxide, catalytic enzymes, or nanoceria and produce oxygen or reactive oxygen species in the site via chemical reactions.^32−35^ Nanoparticles capable of carrying oxygen directly into the site are also being investigated.^36−38^

Hypoxia in the bone marrow and tumors is persistent, requiring continuous and sustained oxygen production for biological effect. Therefore, there is a need to develop a system that can produce oxygen locally and with adjustable kinetics to suit the tumor. We have previously demonstrated that encapsulating manganese dioxide (MnO_2_) NPs in poly(lactic-*co*-glycolic acid) (PLGA) modifies oxygen generation kinetics, reverses hypoxia, and improves NK cell killing of cancer spheroids.^32^ In this study, we investigate how the hydrophobicity of the polymer influences oxygen production kinetics and impacts hypoxia reversal. We demonstrate that long-term oxygen production is necessary for reversing immune suppression. In a bone metastases murine model, the polymer-encapsulated NPs oxygenate the bone metastases lesions, ultimately enhancing the survival of the mice.

## Materials and Methods

### Materials

Poly(d,l-lactide-*co*-glycolide) (PLGA; 50:50, inherent viscosity of 0.95–1.20 dL/g), poly(d,l-lactide-*co*-glycolide) (PLGA; 75:25, inherent viscosity of 0.80–1.20 dL/g), and poly(l-lactide) (PLA, inherent viscosity of 0.90–1.20 dL/g) were purchased from Evonik (Birmingham, AL). Potassium permanganate, chloroform, 30% hydrogen peroxide (H_2_O_2_), agarose (Low-EEO/Multi-Purpose/Molecular Biology grade), Corning RPMI 1640 Medium (Mod.) 1X with l-glutamine, Gibco Trypsin-EDTA (0.25%), Promega CellTiter 96TM AQueous One Solution Cell Proliferation Assay (MTS), poly(vinyl alcohol) (PVA; 80% hydrolyzed, MW = 9,000–10,000), sucrose, and paraformaldehyde were purchased from Sigma-Aldrich (St. Louis, MO). LIVE/DEAD viability/Cytotoxicity Kit for mammalian cells, Alexa Fluor 594 NHS Ester (Succinimidyl Ester), and Image-iT Green Hypoxia Reagent were purchased from Thermofisher (Waltham, MA). HO-PEG-5000-NHS was purchased from Sigma-Aldrich. All cell lines used in this study were purchased from ATCC (Manassas, VA). GeneJet RNA isolation kit for mRNA extraction was obtained from VWR (Radnor, PA). SuperScript III First-Strand Synthesis System used for cDNA synthesis was purchased from Thermofisher (Waltham, MA). AriaMx Real-time PCR System and Ultra-Fast SYBR Green Low ROX QPCR Master Mix were purchased from VWR (Radnor, PA).

### Formulation of MnO_2_ NPs, PEG-MnO_2_ NPs, PLA-MnO_2_, and PLGA-MnO_2_ NPs

As previously described, manganese dioxide (MnO_2_) NPs were synthesized by reducing potassium permanganate with poly(allylamine hydrochloride) (PAH).^39,40^ Briefly, 60 mg of KMnO_4_ was dissolved in 18 mL of ultrapure water (DiH_2_O), to which 60 mg of PAH in 2 mL of DiH_2_O was added with continuous mixing on a magnetic stirrer. The mixture was allowed to react for 30 min, and the formed particles were recovered by centrifugation at 4,000*g* using Amicon Ultra-15 centrifuge tubes (MW cutoff = 100,000 Da, Sigma-Aldrich). The recovered NPs were washed twice with DiH_2_O to remove unreacted KMnO_4_ and PAH.

The synthesized MnO_2_ NPs were encapsulated into PLGA polymers with different hydrophobicity by a modified double emulsion solvent evaporation method.^41,42^ The polymers tested were 50:50 PLGA, 75:25 PLGA, and PLA, where the ratio is defined as lactic acid:glycolic acid to produce 50:50 PLGA-MnO_2_ NPs, 75:25 PLGA-MnO_2_ NPs, and PLA-MnO_2_ NPs, respectively. For encapsulation, a primary emulsion was produced by sonicating 3 mg of MnO_2_ NPs in 200 μL DiH_2_O in 1 mL of chloroform containing 30 mg of PLGA at 38% amplitude (Qsonica Q500) for 1 min on ice. The primary emulsion was added to 12 mL of 3% PVA, vortexed, and sonicated at 38% amplitude (Qsonica Q500) for 3 min on ice with a 10 s pause every minute. The emulsion was stirred at 500 rpm on a magnetic stirrer in a fume hood for a minimum of 4 h to evaporate the chloroform. The formed polymer-encapsulated MnO_2_ NPs were recovered by ultracentrifugation at 45,100*g* for 30 min (Beckman Coulter Optima MAX-XP). After each ultracentrifugation step, the recovered NPs were washed three times with DiH_2_O with resuspension and sonication. After the last washing step, 3% sucrose was added to the NP suspension as a cryopreservative, which was then frozen and lyophilized for 48 h. The lyophilized NPs were stored at −80 °C before use. Control PLGA NPs were generated similarly but used 200 μL DiH_2_O for generating the primary emulsion rather than the 3 mg of MnO_2_ NPs.

For fluorescently labeled NPs, the MnO_2_ NPs were reacted with Alexafluor 594-NHS before their encapsulation into the different PLGA polymers.

Polyethylene-glycol-stabilized MnO_2_ (PEG-MnO_2_) NPs were formed by conjugating activated polyethylene glycol (PEG; HO-PEG-5000-NHS) to the amine groups of the PAH on the MnO_2_ NPs in a 1:1 weight ratio.^32^ In a typical reaction, 10 mg of MnO_2_ NPs was mixed with 10 mg of PEG-NHS in a final volume of 10 mL and reacted for 1 h. The PEG-stabilized NPs were recovered and washed by centrifugation using an Amicon centrifuge filter at 4,000*g*.

### Characterization of NPs

The hydrodynamic diameters and zeta potentials of the formulated NPs (1.0 mg/mL) were determined by dynamic light scattering (DLS; Zetasizer Nano, Malvern) in ultrapure water at room temperature using a refractive index of 1.333 and viscosity of 0.933. The size distribution and morphology of the PLGA-MnO_2_ NPs were confirmed by transmission electron microscopy (TEM) using a JEOL 1200EX. Prior to imaging, the NPs were stained with uranyl acetate for contrast. The percent composition of the MnO_2_ NP after PEGylation or encapsulation into the polymer was determined by inductively coupled plasma mass spectrometry (ICP-MS) analysis. The samples were digested with trace-metal grade nitric acid with heating at 90 °C and diluted with 1% nitric acid before ICP-MS analysis.

### Oxygen Production Kinetics of NPs

The kinetics of oxygen production by the different NP formulations in the presence of H_2_O_2_ was evaluated by adding 50 μg equivalent of Mn of NPs to 10 mL of 0.1 M H_2_O_2_ with continuous stirring. Oxygen levels were measured continuously for 6 h using a dissolved oxygen probe (Thermo Scientific Orion Star) and compared to a baseline oxygen level in DI water and the spontaneous decomposition of H_2_O_2_. The reaction was allowed to continue for another set of samples, and the dissolved oxygen level was measured with the probe after 24 h.

### Cell Culture

All cell lines were purchased from the American Type Culture Collection (ATCC, Manassas, VA). 4T1 and MCF-7 cells were limited for usage within the first 20 passages from the originally purchased vial from ATCC to control for genomic drift due to instability. MCF-7 cells were maintained in DMEM media supplemented with 10% (v/v) heat-inactivated fetal bovine serum (FBS) and 1% penicillin-streptomycin. 4T1 cells were maintained in RPMI media supplemented with 10% (v/v) heat-inactivated fetal bovine serum (FBS) and 1% penicillin-streptomycin. The medium was changed every 2–3 days, and cells were passaged when 65–80% confluent. NK-92 Cells were maintained in l-glutamine containing RPMI supplemented with 20% (v/v) FBS, 1% MEM nonessential amino acid, 1% sodium pyruvate, and 100 units/mL of interleukin (IL) 2. Cells were maintained in a 5% CO_2_ incubator at 37 °C. The MCF-7 spheroids were formed by adding 10,000 cells to 96-well plates precoated with 1% agarose, followed by centrifugation at 1000 rpm. The cells were maintained undisturbed for 72 h in an incubator at 5% CO_2_ and 37 °C to form the spheroids.

### In Vitro Evaluation of Biocompatibility of NPs

The cytotoxicity of the NPs to breast cancer cells was evaluated in two- and three-dimensional (2D/3D) cell cultures. For the 2D culture cytotoxicity, MCF-7 cells were seeded in a 96-well plate at 10,000 cells/well and allowed to adhere overnight. The media was replaced with 100 μL of complete media containing different concentrations of NPs and incubated with the cells for 24 h. After incubation, the NP-containing media was removed, and the cells were washed three times with PBS. The metabolic activity of the cells was measured using the Promega CellTiter 96 AQueous One Solution Cell Proliferation Assay following the manufacturer’s instructions. Since bare MnO_2_ NPs aggregate in media, PEG-MnO_2_ NPs with a similar size as the MnO_2_ NP and possessing a cationic charge were used to ascertain the toxicity of nonencapsulated NPs. The resulting formazan mixture’s absorption was measured using a UV–vis Spectrometer at 490 nm. Results were expressed as the percent viability of NP-treated cells relative to untreated cells under the same conditions.

The toxicity of the NPs to MCF-7 spheroids was evaluated by the LIVE/DEAD Viability/Cytotoxicity Kit (ThermoFisher). Spheroids were treated with different concentrations of the NP formulations for 24 h, after which they were washed with PBS three times and incubated with 4 μM ethidium homodimer and 2 μM calcein-AM for 30 min at room temperature. Spheroids were washed three times with PBS and analyzed via confocal microscopy at 488/520 and 528/633 ex/em (Molecular devices Image Express Confocal Microscope). Relative toxicity was measured by comparing the ethidium fluorescence of the NP-treated spheroids to that of the untreated control.

### In Vitro Evaluation of Hypoxia Reduction by NPs

The ability of MnO_2_ NPs to decrease hypoxia was evaluated in 3D spheroids (Figure S1A). MCF-7 spheroids were incubated with 100 μg/mL of the different NPs formulations in complete media for 2, 6, and 24 h. After incubation with the NPs, the spheroids were transferred into an optically clear U-bottom 96-well plate and incubated with ImageIT Hypoxia Green Stain (2.5 μM) for 45 min. The stained spheroids were evaluated by fluorescent microscopy at 488/520 ex/em (Biotek Lionheart FX Automated Microscope). ImageJ v1.53i was used to quantify the fluorescence intensity, normalizing the signal of the treated groups to the control.

### Cellular Uptake of NPs

Spheroids were incubated with 100 μg/mL of fluorescent-labeled NPs for 24 h, washed with PBS, and fixed in 4% PFA for 1 h. Spheroids were counterstained with Hoescht prior to imaging (Figure S1B).

### Functional Assay for NK Cell Activity

MCF-7 spheroids were incubated with 100 μg of the different NPs formulations in complete media for 2, 6, and 24 h. The pretreated spheroids were coincubated with 20,000 NK-92 cells in OPTI-MEM for 6 h, after which the media was collected. Cancer cell killing was evaluated by lactate dehydrogenase assay (CytoTox 96 Non-Radioactive cytotoxicity assay, Promega) following the manufacturer’s protocol.

### Fluorescent Imaging

MCF-7 spheroid uptake experiments were imaged with a Lionheart LX Automated Microscope (BioTek, Winooski, VT). The Lionheart LX microscope has 4 LED/filter cubes corresponding to DAPI, GFP, Texas Red, and Cy7 and is controlled using BioTek’s Gen 5 software. Confocal microscopy for LIVE/DEAD imaging was performed by using a Molecular Devices ImageXpress Microscope confocal microscope with a Lumencor light engine as the laser source.

### RT-PCR Analysis

The mRNA from spheroids was isolated using a GeneJet RNA isolation kit following the manufacturer’s protocol 24 h postincubation with NPs. At least 90 spheroids were pooled per experiment to ensure a high mRNA yield. The cDNA library was generated using a SuperScript III First-Strand Synthesis System from Thermofisher. RT-PCR was performed using an AriaMx Real-time PCR System and Ultra-Fast SYBR Green Low ROX QPCR Master Mix using the primers listed in Table 1.

**Table 1:** Genes of Interest for RT-PCR

Gene	Forward Primer	Reverse Primer
HIF-1α	TATGAGCCAGAAGAACTTTTAGGC	CACCTCTTTTGGCAAGCATCCTG
PDL1	TGCCGACTACAAGCGAATTACTG	CTGCTTGTCCAGATGACTTCGG
MHC1	CGGCTACTACAACCAGAGCGAG	AGGTCCTCGTTCAAGGCGATGT
GAPDH	GTCTCCTCTGACTTCAACAGCG	ACCACCCTGTTGCTGTAGCCAA
CD73	AGTCCACTGGAGAGTTCCTGCA	TGAGAGGGTCATAACTGGGCAC
TGFβ	TACCTGAACCCGTGTTGCTCTC	GTTGCTGAGGTATCGCCAGGAA

### In Vivo Bone Metastases Model

Female 6–8 week-old BALB/c mice were purchased from Charles River Laboratory. The animals were allowed to adjust in a vivarium for 1 week before experimentation. They were cared for according to protocols approved by the Texas A&M University Animal Care and Use Committee. Animals were randomly assigned to treatment groups and analyzed by experimenters blinded to the treatment groups. Mice were injected with 50,000 4T1 cells via the intracaudal artery to generate bone metastases. Mice were used for further studies 1 week after the 4T1 injection.

### Evaluation of the NP’s Biocompatibility in BALB/c Mice

Female BALB/c mice were injected with 3 doses of 50:50 PLGA-MnO_2_ NPs subcutaneously at 4-day intervals at doses of 2.5 or 10 mg/kg, and their weights were monitored continuously. At 35 days, the mice were euthanized, and blood was collected. A comprehensive metabolic panel of the animals’ blood was performed to assess the animals’ liver and kidney health.

### Tissue Preparation

Mouse tissue samples were isolated and fixed in formalin. The metastases-bearing hindlimbs were demineralized in 14% EDTA in PBS for 5 days, washed in PBS, and then kept in 30% sucrose. The samples were then frozen in Tissue-Tek O.C.T. Compound at −80 °C, and then 10 μm slices were prepared via cryostat. Samples were stored at 4 °C until use.

### Antibody Staining of Histology Slides

Tissue slices on slides were permeabilized in PBS containing 0.1% Triton X-100 (PBST) for 30 min, blocked with 1% serum, and then stained with 1:100 diluted antibodies HIF-1α (NB100–134SS, Novus Biological, Littleton, CO) and FoxP3 (NB100–39002, Novus Biological, Littleton, CO). The samples were washed in PBST three times and then incubated with FITC-tagged goat antirabbit secondary antibody (Novus Biological, Littleton, CO) for 1 h at room temperature. The histology slides were counterstained with Hoescht 33258 dye for 15 min, washed three times with PBST, and analyzed with fluorescent microscopy. ImageJ version 1.53t was used to quantify the fluorescence intensity.

### Biodistribution of NPs In Vivo

The biodistribution of Alexa Fluor 594-tagged 50:50 PLGA-MnO_2_ NPs was assessed using an IVIS Spectrum Imaging System with an ex/em of 590/600. Animals were injected subcutaneously with 5 mg of 50:50 PLGA-MnO_2_ NPs resuspended in 50 μL of PBS. After 24 h, animals were euthanized and perfused with 10 mL of PBS containing 0.1% heparin. Tissues were collected, formalin-fixed, and sectioned. Histology slides were analyzed using fluorescent microscopy.

### Survival Study

Following 1 week of incubation, animals were given 3 subcutaneous injections of 50:50 PLGA-MnO_2_ NP treatment every 4 days at 2.5 mg/kg of the animal’s body weight. The end point was a predetermined point set at 90% weight loss or inability to access water or food due to a lack of mobility.

### Statistical Analysis

All statistical analysis was performed with GraphPad PRISM 9.1.0 (La Jolla, CA). Error bars show standard deviations. Where applicable, 2-way ANOVA with Dunnett’s test was performed to determine statistical significance between groups. Outcomes are denoted as *P < 0.001, #*P* < 0.01, •*P* < 0.05, and ns = no statistical significance.

## Results and Discussion

### NP Formulation and Characterization

**Figure 1:**
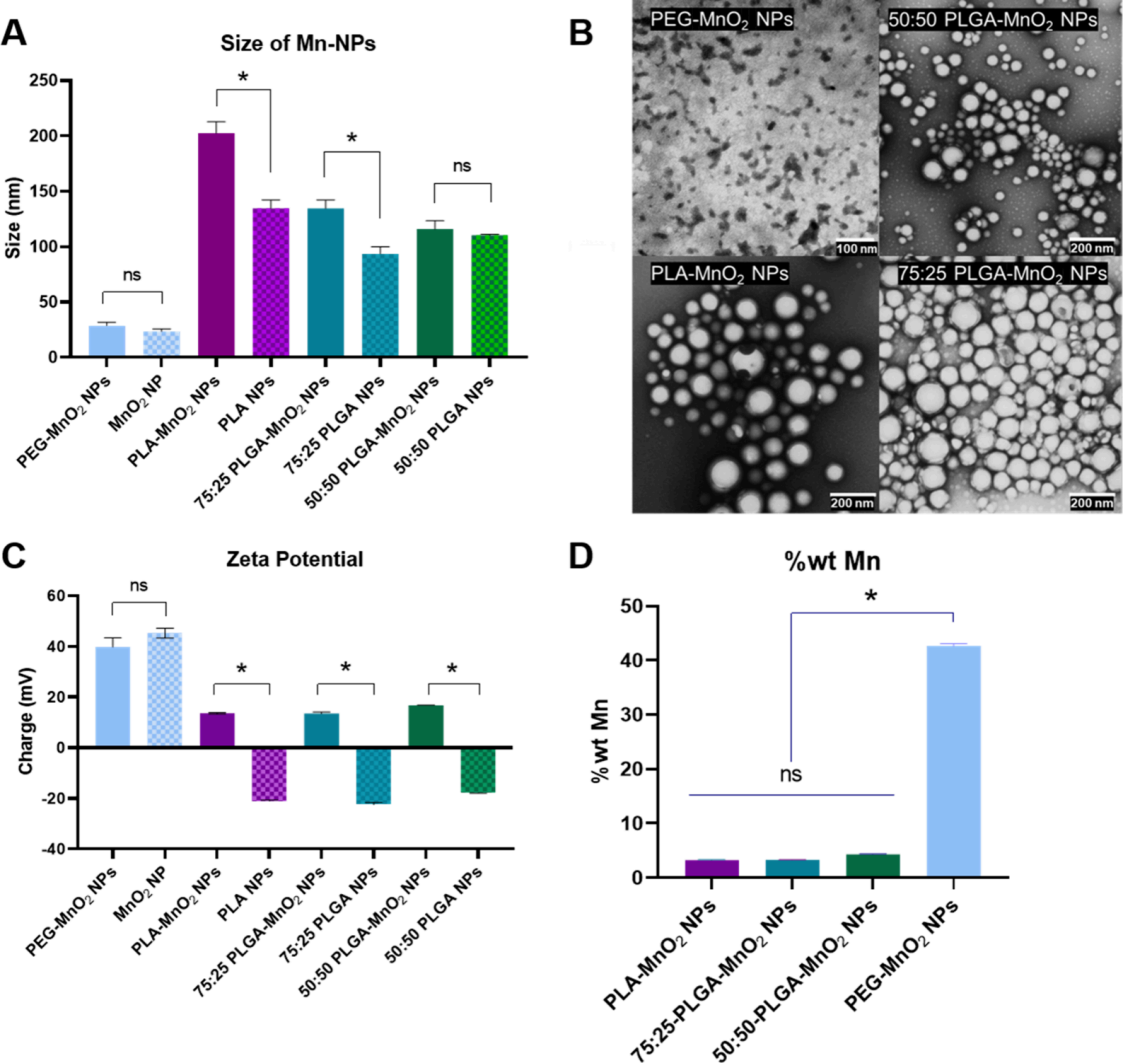
Characteristics of NPs. (A) Dynamic light scattering sizes of different synthesized NPs, PEG-MnO_2_ NPs, PLA-MnO_2_ NPs, 75:25 PLGA-MnO_2_ NPs, and 50:50 PLGA-MnO_2_ NPs. (B) TEM images of PEG-MnO_2_ NPs, PLA-MnO_2_ NPs, 75:25 PLGA-MnO_2_ NPs, and 50:50 PLGA-MnO_2_ NPs showing shape and size distribution. (C) Zeta potential of Synthesized NPs. (D) Weight percentage of manganese in synthesized NPs. Comparisons that are statistically significant are annotated as *P < 0.001, ^#^*P* < 0.01, ^•^*P* < 0.05, and ns = no statistical significance.

Manganese dioxide nanoparticles (MnO_2_ NPs) synthesized by reducing KMnO_4_ with poly(allylamine hydrochloride) had a hydrodynamic diameter of 23.4 ± 4 nm. Since bare MnO_2_ NPs aggregate in phosphate-buffered saline (PBS), they were stabilized with low-molecular-weight polyethylene glycol (PEG), resulting in particles 28.7 ± 3 nm in size. For evaluation of the effect of polymer hydrophobicity on oxygen production kinetics, particles were formulated with polylactic acid (PLA), poly(lactide[75]-*co*-glycolide[25]) (75:25 PLGA), and poly(lactide[50]-*co*-glycolide[50]) (50:50 PLGA) with similar molecular weights by a modified double emulsion solvent evaporation technique. The resulting polymer-formulated NPs without MnO_2_ NPs had hydrodynamic diameters of 134.5 ± 7.6, 93.5 ± 6.3, and 110.2 ± 1 nm for PLA NP, 75:25 PLGA, and 50:50 PLGA, respectively. The increasing lactic acid concentrations created PLA NPs with increased size compared with the PLGA NPs due to the presence of methyl side chains. However, 75:25 PLGA NPs were smaller than the 50:50 PLGA NPs, possibly due to additional van der Waals forces in the methyl side chains combined with the flexibility of the glycolic acid groups. The encapsulation of bare MnO_2_ NPs increased their hydrodynamic size in proportion to the lactic acid content of the polymer. While the hydrodynamic size of the MnO_2_-NPs-encapsulated 50:50 PLGA NPs was 5% larger than the comparable control, the differences in the hydrodynamic size of MnO_2_-encapsulated PLA NPs and 75:25 PLGA NPs were 51% and 44% larger than their respective controls (Figure 1A). Repulsion between the hydrophobic lactic acid side chains and the hydrophilic/ionic MnO_2_ NPs increases with the increasing hydrophobicity of the polymer, leading to larger particle sizes. NPs containing glycolic acid mitigate the increase in size through the flexibility and hydrophilicity of the glycolic acid chains. Transmission electron microscopy (TEM) showed that the resulting NPs were spherical and confirmed their sizes (Figure 1B).

The polymeric particles not loaded with MnO_2_ NPs had negative zeta potential ranging from ∼ −22 mV to ∼ −17 mV. Loading the MnO_2_ into the particles reversed the zeta potential to 13.6 ± 0.3 mV, 13.5 ± 0.5 mV, and 16.7 mV for the PLA-MnO_2_ NPs, 75:25 PLGA-MnO_2_ NPs, and 50:50 PLGA-MnO_2_ NPs respectively (Figure 1C).

Since the double emulsion-solvent evaporation technique encapsulates the hydrophilic MnO_2_ into the hydrophobic polymer, it was investigated whether the polymer hydrophobicity affects loading efficiency. ICP-MS measurement of Mn content in a unit mass of NPs shows that 50:50 PLGA-MnO_2_ NPs had Mn content significantly higher than those of the 75:25 PLGA-MnO_2_ NPs and PLA-MnO_2_ NPs (Figure 1D). However, the Mn content between the 75:25 PLGA-MnO_2_ NPs and the PLA-MnO_2_ NPs was not statistically significant.

### Polymer Hydrophobicity Affects the Oxygen-Production Kinetics of Polymer-Encapsulated MnO_2_ NPs

**Figure 2:**
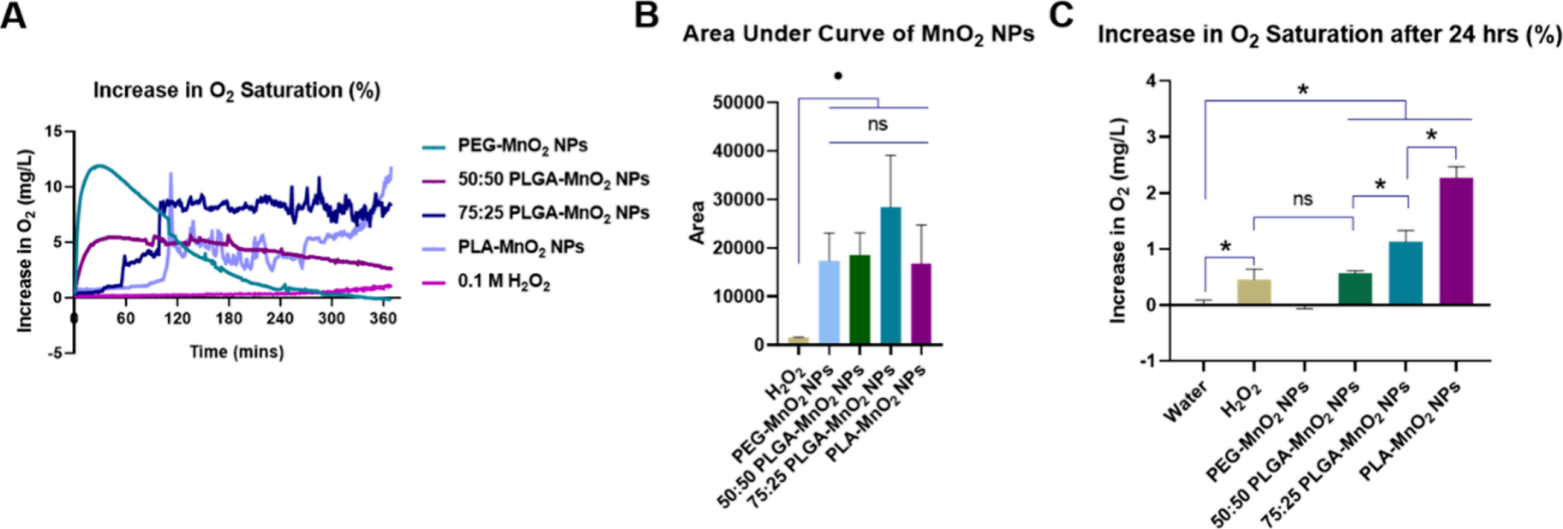
Characteristics of NPs. (A) Increase in oxygen saturation over 4 h of each NP. (B) Area under the curve of oxygenation kinetics to estimate cumulative O_2_ production. (C) Increase in oxygen saturation after 24 h. Statistically significant comparisons are annotated as *P < 0.001, ^#^*P* < 0.01, ^•^*P* < 0.05, and ns = no statistical significance.

Changing the hydrophobicity of the encapsulating polymer alters the reaction kinetics between MnO_2_ and H_2_O_2_. Encapsulating MnO_2_ NPs in 50:50 PLGA results in first-order oxygen production in the presence of H_2_O_2_ compared to the burst oxygen production by pegylated MnO_2_.^32^ These findings were confirmed in the current study, where the easy access of H_2_O_2_ to PEG-MnO_2_ NPs resulted in burst production of O_2_, reaching a maximum increase in O_2_ saturation of 11.8 mg/L within 30 min before gradually decreasing to baseline oxygen saturation in 4 h. The 50:50 PLGA-MnO_2_ NPs showed a gradual increase in oxygen production and increased the level of the level of the O_2_ saturation by 50%, maintaining this level for several hours (Figure 2A). Unlike the PEG-MnO_2_ NPs, the 50:50 PLGA-MnO_2_ NPs, O_2_ saturation was increased by 5.5 mg/L even after 4 h. Relative to the 50:50 PLGA-MnO_2_ NPs, the oxygen production from the PLA-MnO_2_ NPs and 75:25 PLGA-MnO_2_ NPs was delayed, with the time for a significant increase in O_2_ saturation dependent on the polymer hydrophobicity (Figure 2A). After an initial 60 min delay in O_2_ production, the 75:25 PLGA-MnO_2_ maintained the O_2_ tension at a level higher than that observed for the 50:50 PLGA-MnO_2_ for 5 h. The PLA-MnO_2_ NPs showed a progressive increase in the level of the O_2_ with time, reaching levels higher than the other NPs by the sixth hour of the experiment.

The O_2_ production plots for PLA-MnO_2_ NPs and 75:25 PLGA-MnO_2_ NPs also showed an intermittent burst of O_2_ production that is absent for the 50:50 PLGA MnO_2_ NPs. The encapsulated MnO_2_ may form clusters in pockets of the polymer due to their immiscibility, and these clusters of MnO_2_ could produce a burst of O_2_ when exposed to H_2_O_2_. Cumulatively, the PEG-MnO_2_ NP, 50:50 PLGA-MnO_2_ NP, 75:25 PLGA-MnO_2_ NPs, and PLA-MnO_2_ NPs produced similar amounts of O_2_ over the 6 h (Figure 2B).

Because tumors have increased acidity, which can affect the kinetics of O_2_ production, the O_2_ production kinetics was performed at pH 6.8. The reaction of MnO_2_ with H_2_O_2_ to produce oxygen (MnO_2_ + H_2_O_2_ + 2H^+^ → O_2_ + Mn^2+^ + 2H_2_O) is promoted by available protons, which increase with decreasing pH, thus increasing the reaction kinetics. The available proton increased the rate of O_2_ production, especially in the initial two hours, but showed similar levels at pH 7.4 afterward (Figure S2). As expected, the effect of pH was more pronounced on the PEG-stabilized NPs than the polymer-encapsulated NPs due to reduced access to the MnO_2_.

After 24 h, the increase in oxygen saturation maintained by the PLA-MnO_2_ NPs was higher (25%) than the 50:50 PLGA-MnO_2_ and 75:25 PLGA-MnO_2_ NPs (7% and 12%, respectively) (Figure 2C). At 24 h, the O_2_ saturation for the PEG-MnO_2_ NPs was at baseline levels. This suggests that the PEG-MnO_2_ NPs cannot maintain an environment of high oxygen tension, and the PLA-MnO_2_ NPs can continually generate oxygen.

### Hydrophobicity of Encapsulating Polymer Affects the Cytotoxicity of PLGA-MnO_2_ NPs

**Figure 3:**
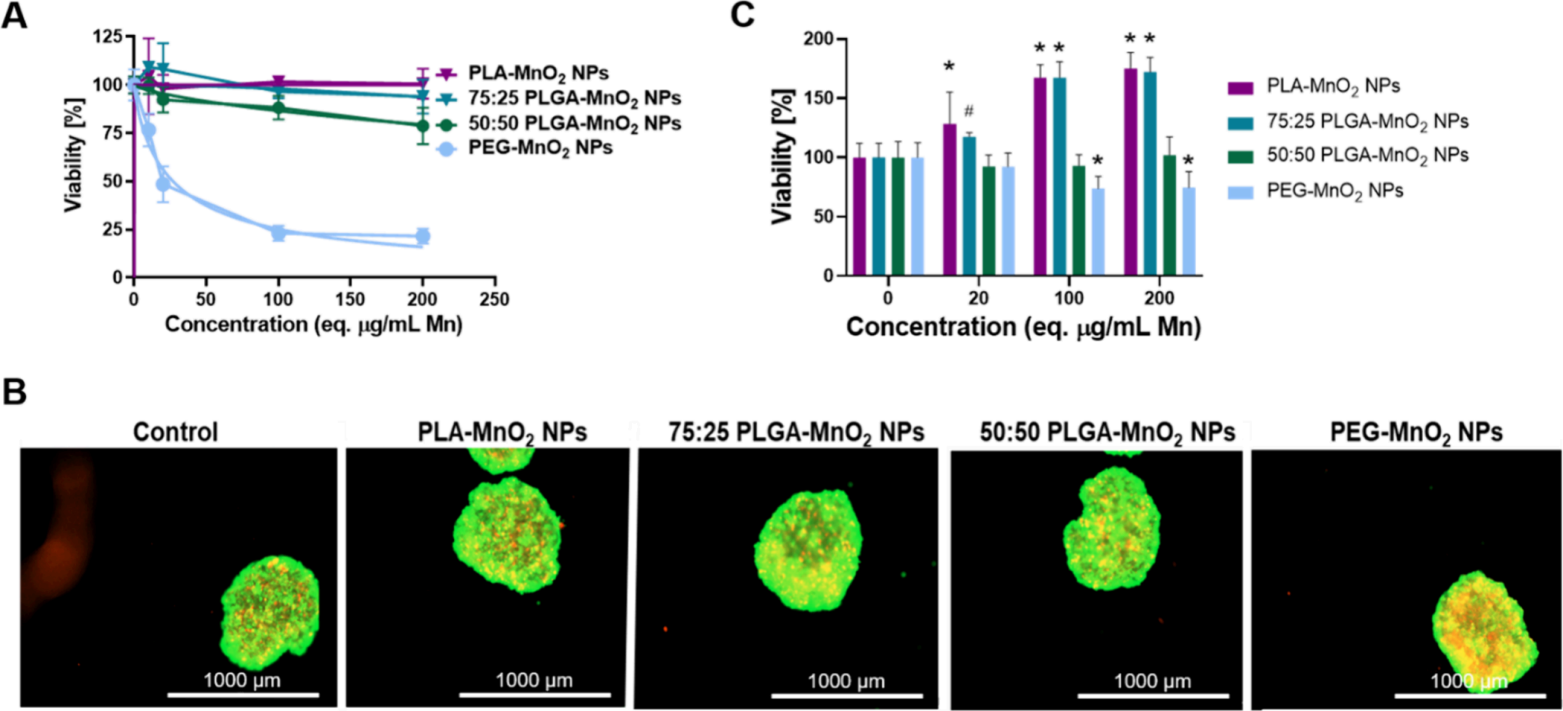
Biocompatibility of NPs in MCF-7 cells. (A) Viability of MCF-7 cells after exposure to PEG-MnO_2_ NPs, 50:50 PLGA-MnO_2_ NPs, 75:25 PLGA-MnO_2_ NPs, and PLA-MnO_2_ NPs in 2D culture measured using MTS assay. (B) Representative images of LIVE/DEAD stain on MCF-7 spheroids treated with 200 μg/mL Mn equivalent of NPs. (C) Quantification of LIVE/DEAD stain on MCF-7 spheroids to verify viability after incubation with different formulations of MnO_2_ NPs. Comparisons were made between the control (0 μg/mL) group for each set of nanoparticles. Statistically significant treatment groups were denoted with *P < 0.001 and ^#^*P* < 0.01.

The cytotoxicity of PEG-MnO_2_ NPs, 50:50 PLGA-MnO_2_ NPs, 75:25 PLGA-MnO_2_ NPs, and PLA-MnO_2_ NPs was evaluated in 2D and 3D cell cultures. For 2D cytotoxicity, a monolayer of MCF-7 cells was incubated with different NP concentrations for 24 h before the MTS assay. The PEG-MnO_2_ NPs showed higher levels of cytotoxicity with an LD_50_ of 26 μg/mL. For the 50:50 PLGA-MnO_2_ NPs, cell viability was ∼80% after treatment with 200 μg/mL eq. Mn. The PLA-MnO_2_ NPs and 75:25 PLGA-MnO_2_ NPs did not affect cell viability, which remained at 100% at the same concentration (Figure 3A). The differences in toxicity indicated that the encapsulation successfully reduced the Mn toxicity in cells. The rate of Mn release intracellularly could influence toxicity, as the polymer hydrophobicity limits the rate Mn^2+^ is produced when the MnO_2_ reacts with H_2_O_2_ or glutathione.^43^

The effects of these NPs on a 3D model were evaluated in spheroids capable of maintaining a hypoxic core similar to that of tumors. Spheroids generated with 10,000 MCF-7 cells were incubated for 24 h with different concentrations of the formulated NPs, and biocompatibility was analyzed by LIVE/DEAD assay (Figure 3B, C). The ethidium homodimer signal from the spheroids was analyzed to quantify toxicity. The PEG-MnO_2_ had an IC_50_ > 200 μg/mL eq. Mn to the spheroids, significantly higher than observed in the 2D assay. Therapeutics show lower toxicity in 3D environments due to the decreased penetration and other biological functions induced by a supportive extracellular matrix that supports and promotes viability.^44−46^ The 50:50 PLGA-MnO_2_ NPs did not induce toxicity at the highest concentration tested. In contrast, both 75:25 PLGA-MnO_2_ NPs and PLA-MnO_2_ NPs enhanced the viability of the spheroids (Figure 3B, C), likely due to the reversal of hypoxia in the necrotic core of the spheroid, rescuing cells normally restricted by the low oxygen tension.^44−46^

**Figure 4:**
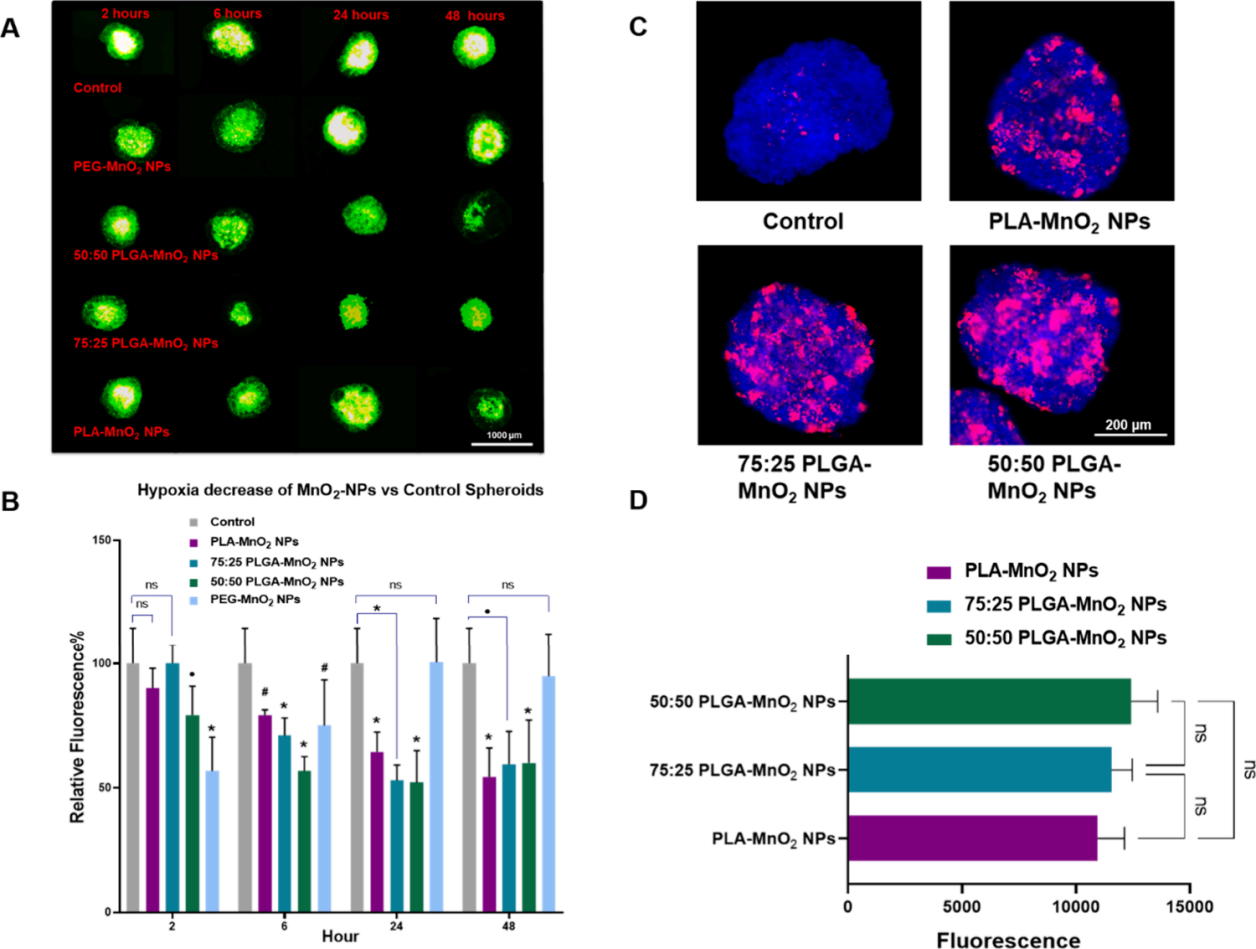
Effects of NPs on MCF-7 spheroids. (A) Representative images of hypoxia levels in PEG-MnO_2_ NPs, 50:50 PLGA-MnO_2_ NPs, 75:25 PLGA-MnO_2_ NPs, and PLA-MnO_2_ NPs treated spheroids measured by hypoxia-sensitive dye ImageIT. (B) Quantification of ImageIT signal by ImageJ in NP-treated spheroids to measure extent of hypoxia reduction. Representative image (C) and quantification (D) of NP uptake in spheroids after 24 h exposure. Spheroids were fixed and washed prior to imaging using confocal microscopy. Comparisons that are statistically significant are annotated as *P < 0.001, ^#^*P* < 0.01, ^•^*P* < 0.05, and ns = no statistical significance.

For the hypoxia-relieving effectiveness of the different MnO_2_ NPs formulations, MCF-7 spheroids were incubated with 100 μg/mL eq. Mn of NPs and the hypoxia level were measured using an ImageIT hypoxia probe at different time points (Figure 4A, B). After 2 h, the PEG-MnO_2_ NPs and 50:50 PLGA-MnO_2_ groups decreased the hypoxia levels in the spheroid by 40% and 20%, respectively, while the 75:25 PLGA-MnO_2_ NP and PLA-MnO_2_ NP showed no effects (Figure 4A, B, S3). After 6 h with the spheroids, all particle formulations reduce hypoxia levels in the spheroids, albeit at different levels. The 50:50 PLGA-MnO_2_ NPs decreased hypoxia by 48%, while the 75:25 PLGA-MnO_2_ NPs, PLA-MnO_2_ NPs, and PEG-MnO_2_ NPs reduced hypoxia by 30%, 21%, and 25%, respectively. The PEG-MnO_2_ NPs, however, did not reduce hypoxia in 24 and 48 h, indicating that the rapid burst of oxygen production is insufficient to reduce hypoxia for long durations. The delayed oxygen production kinetics of the PLA-MnO_2_ NPs translated to their ability to reduce hypoxia at comparable levels to 75:25 PLGA-MnO_2_ NPs and 50:50 PLGA-MnO_2_ NPs after 24 and 48 h (Figure 4A, B). These results support the need for continuous oxygen production in particulate systems to relieve sustained hypoxia in biological systems.

To assess if the differences in the hypoxia control or decrease in toxicity were related to NP interaction with the spheroids, NPs were fluorescently tagged with Alexafluor 594, and their interaction with the spheroids was evaluated by fluorescent microscopy. These studies showed no difference in the interactions of the 50:50 PLGA-MnO_2_ NPs, 75:25 PLGA-MnO_2_ NPs, and PLA-MnO_2_ NPs with the spheroids (Figure 4C, D).

### PLGA-MnO_2_ NPs Improve the Efficacy of Immune Cell Function in Tumor Spheroids

**Figure 5:**
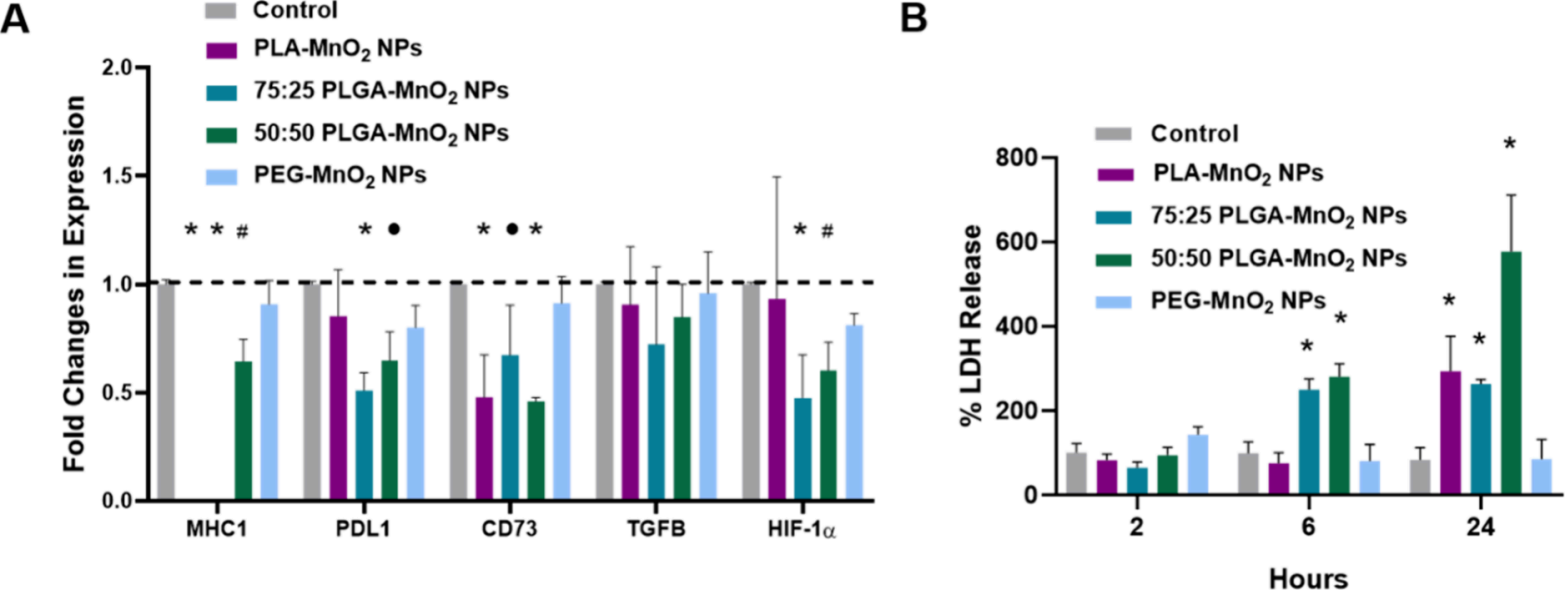
Effects of NPs on MCF-7 spheroids. (A) Fold changes in expression of target genes in NP-treated MCF-7 spheroids at different time points. (B) LDH assay showing the increase in killing efficacy of NK-92 cells on MCF-7 spheroids. Comparisons were made between the control group for each set of nanoparticles. Statistically significant treatment groups were denoted with *P < 0.001, ^#^*P* < 0.01, and ^•^*P* < 0.05.

Hypoxia limits the effectiveness of immunotherapies by promoting the upregulation of immunosuppressive genes.^47^ The decrease in spheroid hypoxia should downregulate the expression of HIF-1a, interfering with immunosuppression. The different kinetics of the PEG-MnO_2_ NPs, 50:50 PLGA-MnO_2_ NPs, 75:25 PLGA-MnO_2_ NPs, and PLA-MnO_2_ NPs might also optimize the time required to reverse the TME effectively. We explored this by treating MCF-7 spheroids with the different NPs for 24 h and analyzing the expression of hypoxia-inducing factor 1-α (HIF-1α), major histocompatibility complex 1 (MHC1), programmed death ligand 1 (PDL1), transforming growth factor beta (TGF-β), and CD73. There was a notable decrease in CD73 expression in PLA-MnO_2_, 75:25 PLGA-MnO_2_, and 50:50 PLGA-MnO_2_ groups. The 75:25 PLGA-MnO_2_ and 50:50 PLGA-MnO_2_ NP groups also showed a decrease in the levels of expression of PD-L1 and HIF-1α (Figure 5A). The decrease in CD73 could lead to improved immune response due to the decrease in the immunosuppressive metabolite adenosine.^48−50^ The faster-reacting PLGA NPs also decreased the level of PD-L1 and HIF-1α expression. The different expression profiles of each type of NP indicate that different kinetics could affect the timeline of hypoxia reversal and thus the reversal of the immunosuppressive phenotype.

Since the NPs showed differences in the degree to which they decreased the expression of several immune-related factors, we investigated how this would impact the killing efficiency of natural killer cells. Pretreating the spheroids for 2 h before adding the NK cells did not impact their cytotoxicity. When NP-pretreated spheroids were coincubated with NK-92 cells, there were substantial improvements in NK cell killing efficacy after 6 h of treatment with both 50:50 PLGA-MnO_2_ and 75:25 PLGA-MnO_2_ NP groups (Figure 5B). After 24 h, however, PLA-MnO_2_ NPs also improved the killing efficiency of NK-92 cells. The pretreatment of spheroids with the 50:50 PLGA-MnO_2_ NPs group enhanced the killing efficiency of NK cells better than the other NPs, possibly due to extended decreased PD-L1 and CD73 expression. PEG-MnO_2_ NPs did not influence the killing efficacy of the NK cells, indicating that sustained hypoxia reversal is essential for restoring immune cell function. Since NK cells are not as dependent on MHC1 recognition for cytotoxic activity, there seemed to be little effect of the decreased MHC1 expression in NK cell-mediated destruction.^51−53^ The 50:50 PLGA-MnO_2_ NPs improved the immune responses across all periods and, thus, were an optimal choice in moving forward into in vivo studies.

### 50:50 PLGA-MnO_2_ NPs Are Biocompatible In Vivo

**Figure 6:**
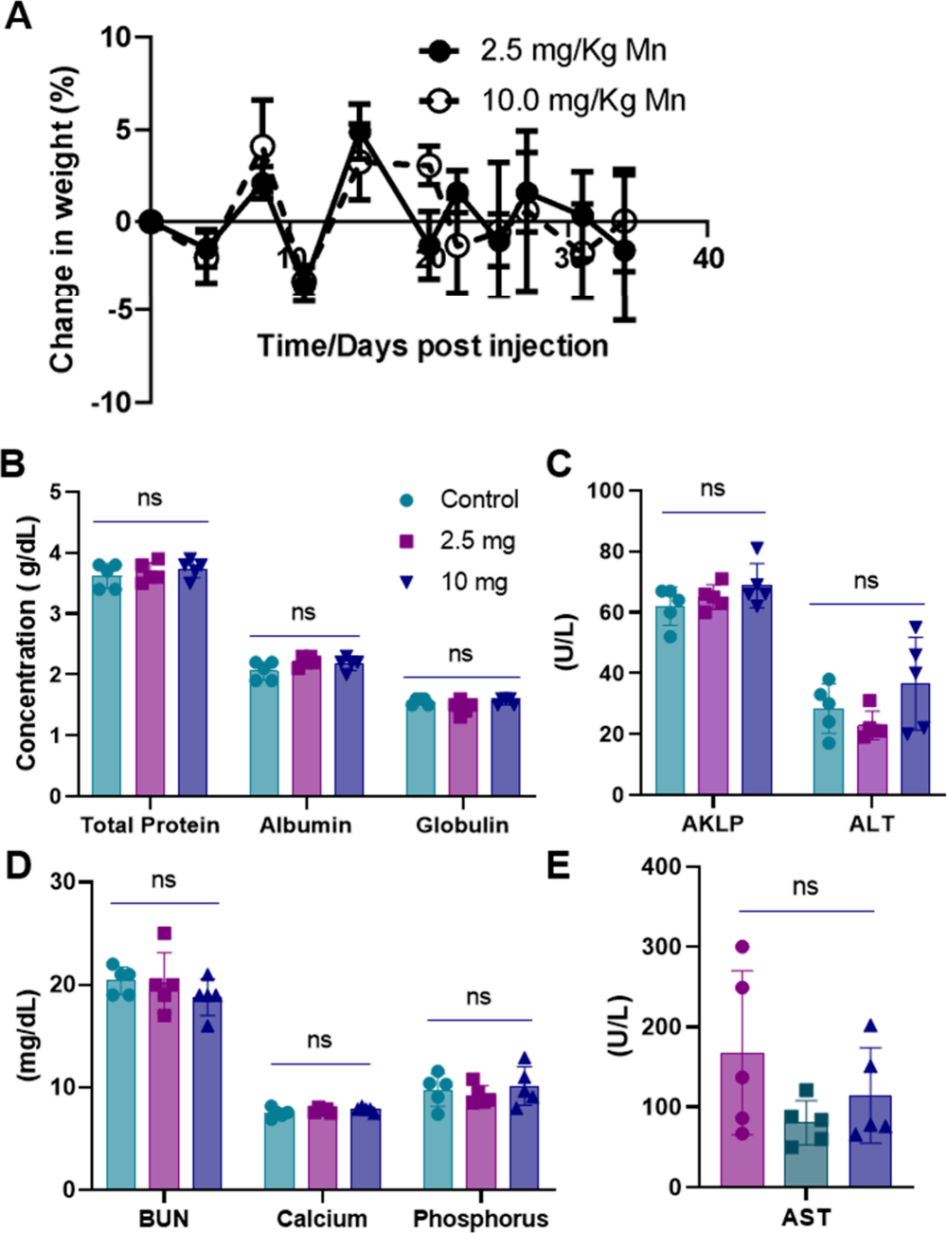
Biocompatibility of NPs in BALB/c mice. (A) Weight change post-NP injection. (B–E) Blood chemistry profile of NP-injected mice of total serum protein, albumin, globulin, bilirubin, calcium, phosphorus, aspartate transaminase (AST), alanine transaminase (ALT), and alkaline phosphatase (AKLP) in the blood of mice 35 days post NP injection.

Because of their superior effects at enhancing the cytotoxicity of NK cells in vitro, the 50:50 PLGA MnO_2_ NPs were evaluated in vivo, initially to ascertain their biocompatibility by injecting naïve BALB/c mice subcutaneously at 2.5 mg/kg of NPs or 10 mg/kg in three doses at four-day intervals. Over 35 days, the animal weight did not decrease significantly, and no abnormal behaviors were detected (Figure 6A). A metabolic panel was performed 35 days after injection to investigate the animal’s health. The animals’ kidney function was evaluated by measuring Blood Urea Nitrogen (BUN), while the levels of aspartate transaminase (AST), alanine transaminase (ALT), and alkaline phosphatase (ALKP) were examined to determine the liver function (Figure 6B–E).^54^ Despite multiple doses, 50:50 PLGA-MnO_2_ NPs did not seem to significantly alter the organ function of the animals, indicating that PLGA encapsulation successfully mitigated potential side effects both in vitro and in vivo.

### 50:50 PLGA MnO_2_ NPs Improve Survival of Bone Metastasis Mice by Reducing Intratumoral Hypoxia in Bone Metastasis Sites

**Figure 7:**
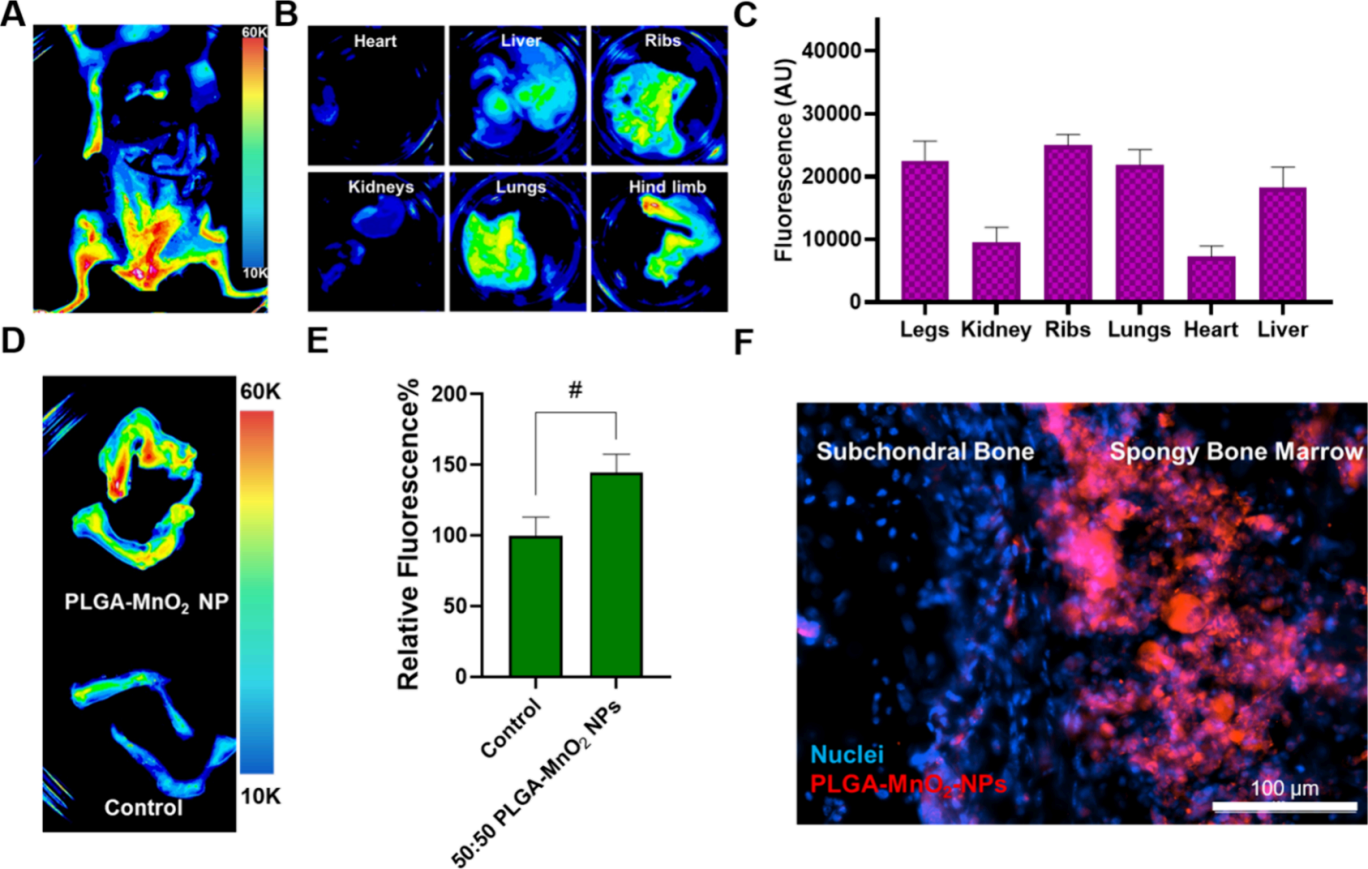
Biodistribution of NPs in BALB/c mice. (A–B) Ex vivo imaging of organ system within the animal showing preferential distribution into the hind limbs. (C) Quantification of fluorescence signal in organs of treated animals. (D) Ex vivo image of hindlimbs of NP-injected and control mice. (E) Quantification of increased NP uptake into the hindlimbs. (F) Fluorescent image showing NP accumulation in spongy bone. Comparisons that are statistically significant are annotated as *P < 0.001, ^#^*P* < 0.01, ^•^*P* < 0.05, and ns = no statistical significance.

Fluorescently labeled nanoparticles were subcutaneously injected into the mice and imaged ex vivo after 24 h. The NPs accumulated in the hind limbs and pelvis of the animal (Figure 7A). Ex vivo imaging confirmed that the NPs have preferential accumulation in the long bones and ribs, common sites of bone metastases (Figure 7B). The NPs have relatively low accumulation in the kidneys, lungs, and liver. The mean NP accumulation was 1.45 times higher in long bones and ribs than in the liver (Figure 7B, C). Furthermore, there was increased NP uptake in the long bones of treated animals versus that of untreated animals (Figure 7D, E). Nanoparticle targeting of bone lesions has historically relied on active targeting with ligands that target the hydroxyapatite of mineralized bone, such as bisphosphonates. We have previously demonstrated that NPs smaller than 120 nm and with surface charge close to neutral accumulate in the bone marrow by taking advantage of the fenestrations in the sinusoidal capillaries of the bone marrow vasculature.^5,59,60^ The present study reinforces these findings and demonstrates that bone accumulation of bioactive NPs can be achieved at sufficient concentrations without active targeting to elicit biological change. These studies show that taking advantage of the physiological characteristics of the tissue vascular ensures nanoparticle accumulation. Since the nanoparticles were slightly positive in charge and smaller, histological sectioning and fluorescent imaging confirmed that the NPs accumulate in the spongy bone where bone metastasizing cancer cells reside (Figure 7F). The spongy bone marrow contains a complex vasculature, making it a common site for metastases. Therefore, it is promising that 50:50 PLGA-MnO_2_ NPs can enter the area, increasing their likelihood to interact with existing tumors.

**Figure 8:**
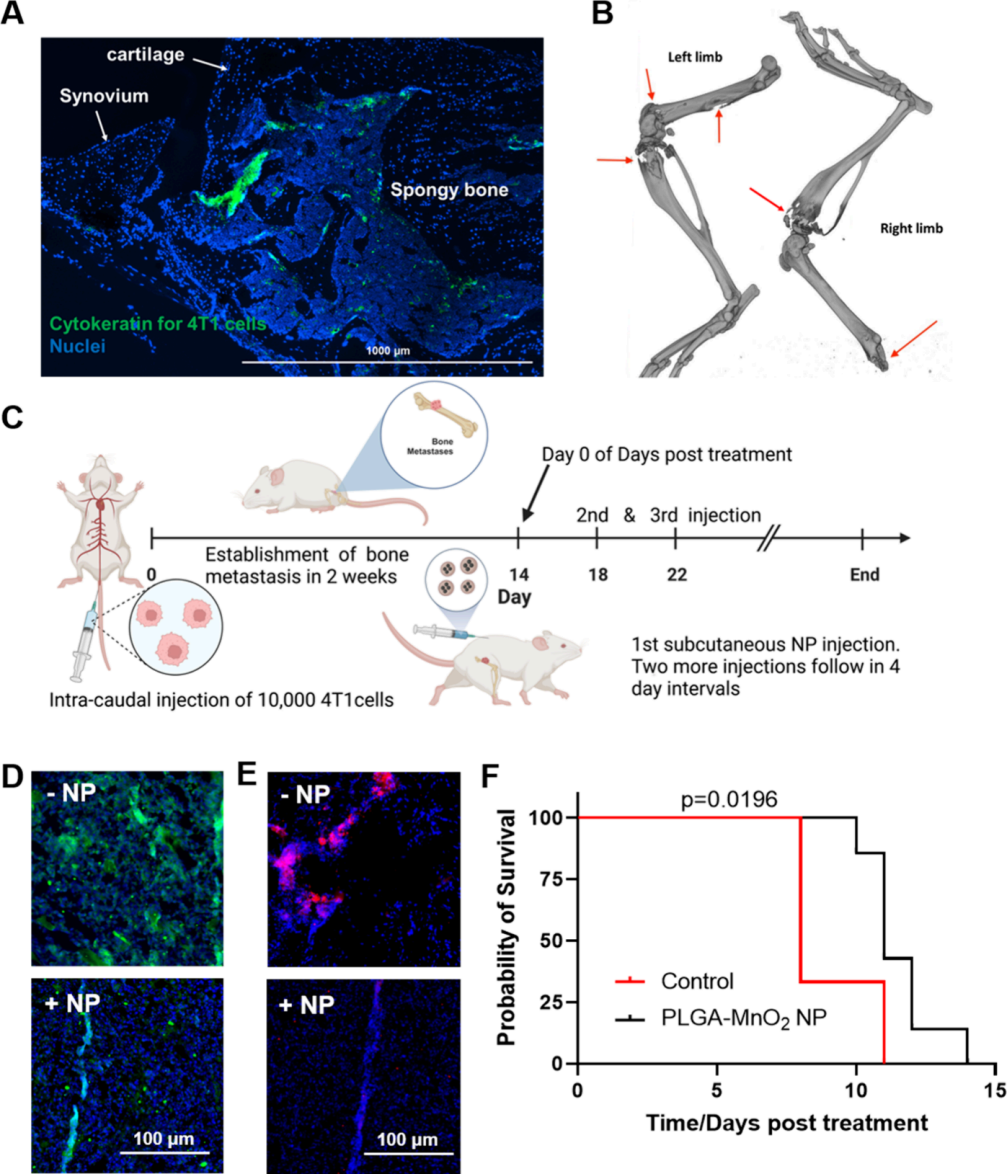
Effects of NPs treatment in BALB/c mice. (A) Immunofluorescent staining of 4T1 cells present in the bone marrow of tumor positive mice. (B) CT imaging of bone desorption. (C) Timeline of treatment of tumor-burdened animals with 50:50 PLGA-MnO_2_ NPs. (D) HIF-1α expression in the bone metastases of the animal. (E) Immunofluorescent staining of FOXP3 for regulatory T cell populations. (F) Survival curves with and without 50:50 PLGA-MnO_2_ NP treatment.

To determine the 50:50 PLGA-MnO_2_ NPs’ capacity to reduce tumor hypoxia in metastases lesions, a bone metastases model that is consistent and repeatable was generated in immune-competent BALB/c mice (Figure S4).^55^ The 4T1 cells were injected via the caudal artery of mice, allowing for first-pass accumulation into the hind limbs. Compared to intracardiac injection of cancer cells to generate bone metastasis, caudal artery injection results in a 100% incidence of bone metastasis in injected mice. Unlike the intraosseous model for inducing bone metastasis, which requires direct cancer cell injection into the tibia, this method does not disrupt the bone tissue during metastasis initiation. The localization of the injected 4T1 cells in bone was verified by immunohistochemistry. The injected cancer cells colonized the spongy bone tissue in the epiphysis (Figure 8A). The epiphysis of long bones is a common site for bone metastasis initiation because of cavities in spongy tissues. The lytic nature of the cancer cells was observed by computed tomography (CT) imaging that showed the rapid progression of the metastasis, showing significant bone loss 2 weeks postinjection of the cells (Figure 8B). In our pilot survival study, PLGA-MnO_2_ NPs (2.5 mg Mn/kg) were injected subcutaneously every 4 days after the establishment of the bone metastases (Figure 8C). 50:50 PLGA-MnO_2_ NP treatment decreased the level of HIF-1α, a key regulator of hypoxic response (Figure 8D). Because the bone environment and metastatic lesions are highly hypoxic, the decrease in the HIF-1α level indicates the ability of the NPs to maintain a high enough oxygen tension in the bone for sustained periods to ensure protein destabilization. The bone marrow harbors highly regulatory T (Treg) cells that maintain immunosuppressive niches in the bone marrow. The number of functional Treg cells increases in bone metastases, worsening the immunosuppression and promoting disease progression.^56−58^ 50:50 PLGA-MnO_2_ NP treatment decreased the regulatory T cell (Treg) levels in the bone metastases lesions (Figure 8E). These results indicate that sufficient NPs reach the bone metastases lesions to mediate immunogenic effects, providing an environment that is supportive of the antitumor function of the immune system. These observations were confirmed by the survival study which showed that the NP-mediated hypoxia reduction alone is sufficient to improve bone-metastasis-bearing mice’s survival. Our study shows that even low doses of the NP system can significantly increase mice survival from a median of 8 to 11 days post-treatment (*p* = 0.0196) (Figure 8F). These results highlight that hypoxia reduction by the 50:50 PLGA-MnO_2_ NPs, even at low doses, improves the survival of mice presenting with established aggressive bone metastasis.

## Conclusion

The study demonstrates that controlled oxygen kinetics can reverse hypoxia and improve immune activity. Increasing the encapsulating polymer’s hydrophobicity can alter oxygen production’s kinetics by MnO_2_-containing NPs and mitigates potential harmful effects. The altered kinetics changed the degree of hypoxia reversal and its effect on immunosuppressive genes. By reversing hypoxia within the spheroid, we showed a decrease in PD-L1 and CD73, resulting in improved NK cell activity. In vivo experiments also showed that 50:50 PLGA-MnO_2_ NPs can reduce hypoxia within the bone metastasis site and improve the survival of bone-metastasis-bearing mice with minor side effects. This work showed that hypoxia reversal via oxygen-producing nanoparticles could improve the survival of patients with bone metastases. The in vivo results offer encouragement that oxygen-producing NPs such as the PLGA-MnO_2_ improve immune responses against bone metastasis, potentially paving the way for synergistic treatments with other therapeutics. Thus, future experiments will focus on the degree of immune activation as well as using PLGA-MnO_2_ NPs as a codrug.

## Supporting Information Available

A scheme for the in vitro hypoxia and uptake experiments; the effects of pH on PLGA-MnO_2_ NP versus PEG-MnO_2_ NP activity; overlaid images of the stained spheroids and the brightfield images to demonstrate the decrease in size of the hypoxic core following NP treatment; scheme of how the bone metastases were generated in the BALB/c mice following 4T1 cell injection (PDF)

## Notes

The schemes presented were created using Biorender.com.
