# Supplementary material for: Optimizing Oxygen-Production Kinetics of Manganese Dioxide Nanoparticles Improves Hypoxia Reversal and Survival in Mice with Bone Metastases
Source: Mol Pharm. 2024 Feb 16;21(3):1125–36. doi: 10.1021/acs.molpharmaceut.3c00671 (PMC10979454; doi:10.1021/acs.molpharmaceut.3c00671)
Supplement: Supplementary file 1 — mp3c00671_si_001.pdf [file mp3c00671_si_001.pdf]

Optimizing oxygen-production kinetics of manganese dioxide nanoparticles improves hypoxia reversal and survival in mice with bone metastases

Authors: David A. Murphy, Daniela Osteicochea, Aidan Atkins, Caitlin Sannes, Zachary McClarnon, and Isaac M. Adjei*

Department of Biomedical Engineering, Texas A&M University, College Station, TX 77843, USA

*Corresponding author:

Isaac M. Adjei, Ph.D.

Department of Biomedical Engineering

Texas A&M University

College Station, TX 77843

Phone: 979-458-2536

Fax: 979-845-4450

Email: adjeii@tamu.edu

Supplementary Information

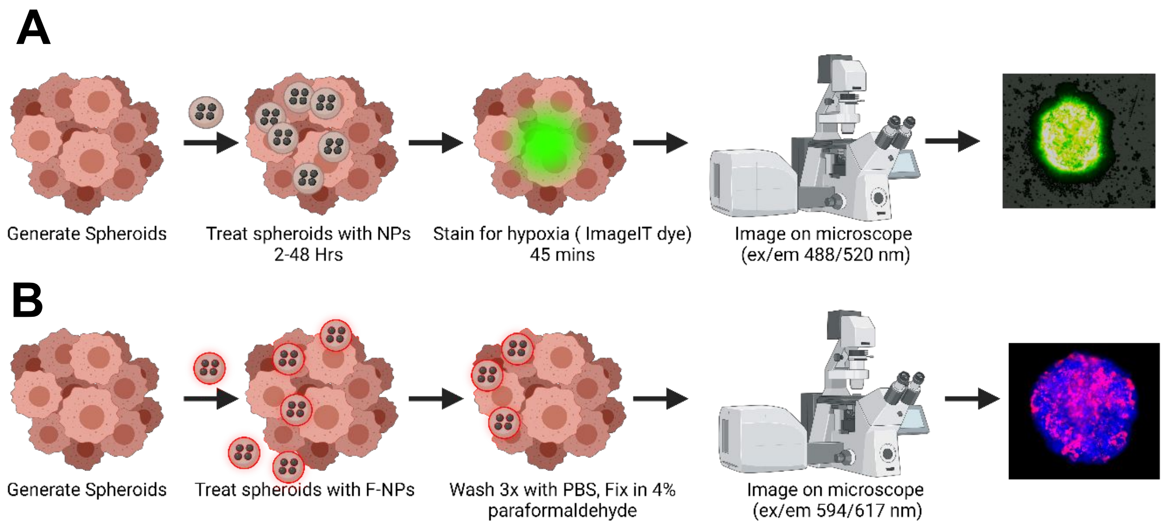

Figure S1: Schemes for imaging spheroid hypoxia (A) and NP uptake (B)

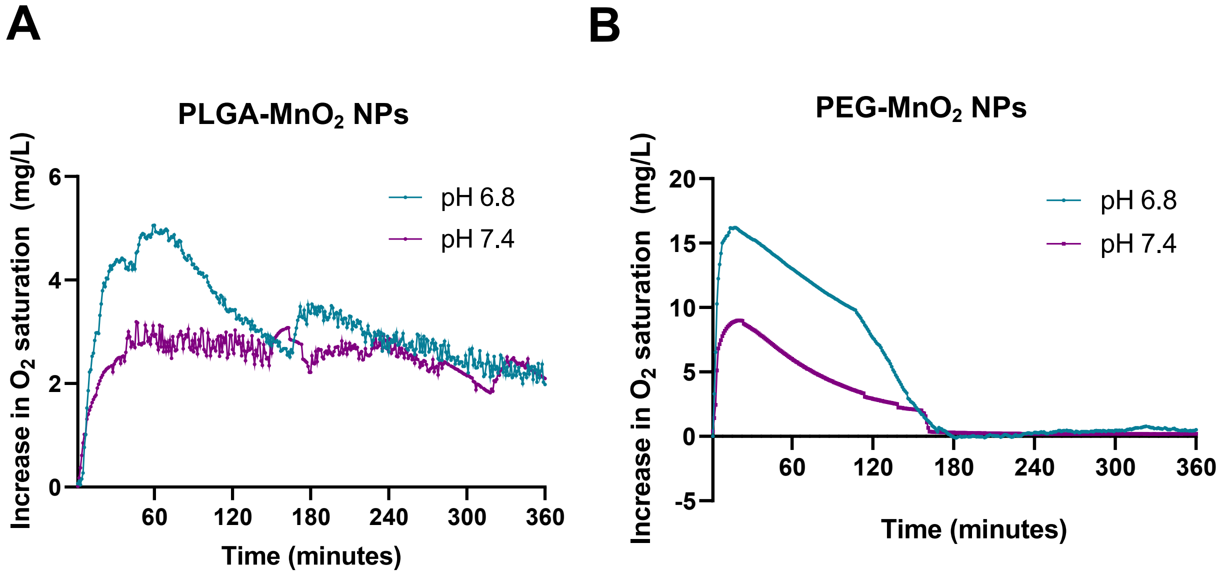

Figure S2: Increase in O_2 saturation in different pHs for PLGA-MnO_2 NPs (A) and PEG-MnO_2 NPs (B)

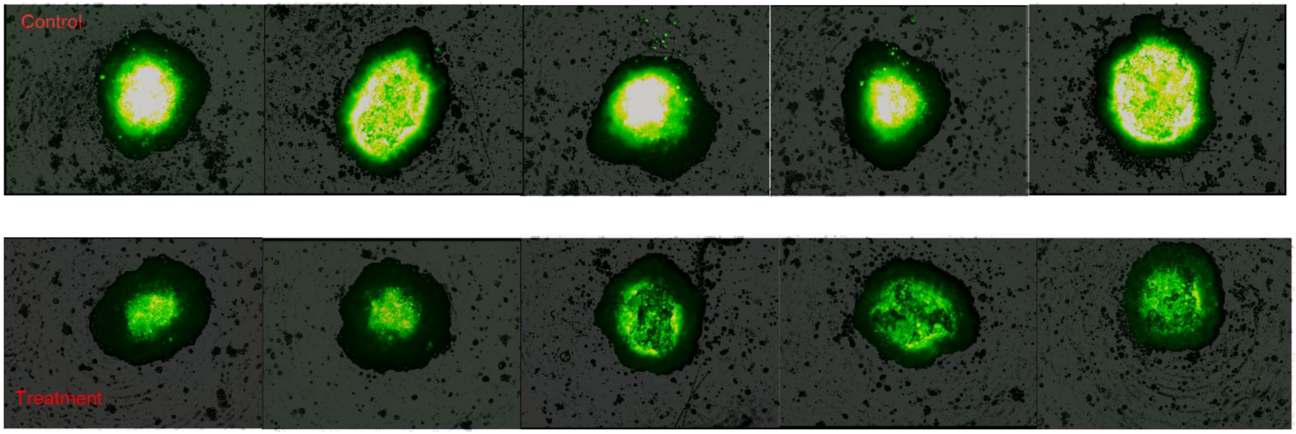

Figure S3: Overlay of brightfield and fluorescent images showing decreasing size of hypoxic core after nanoparticle treatment.

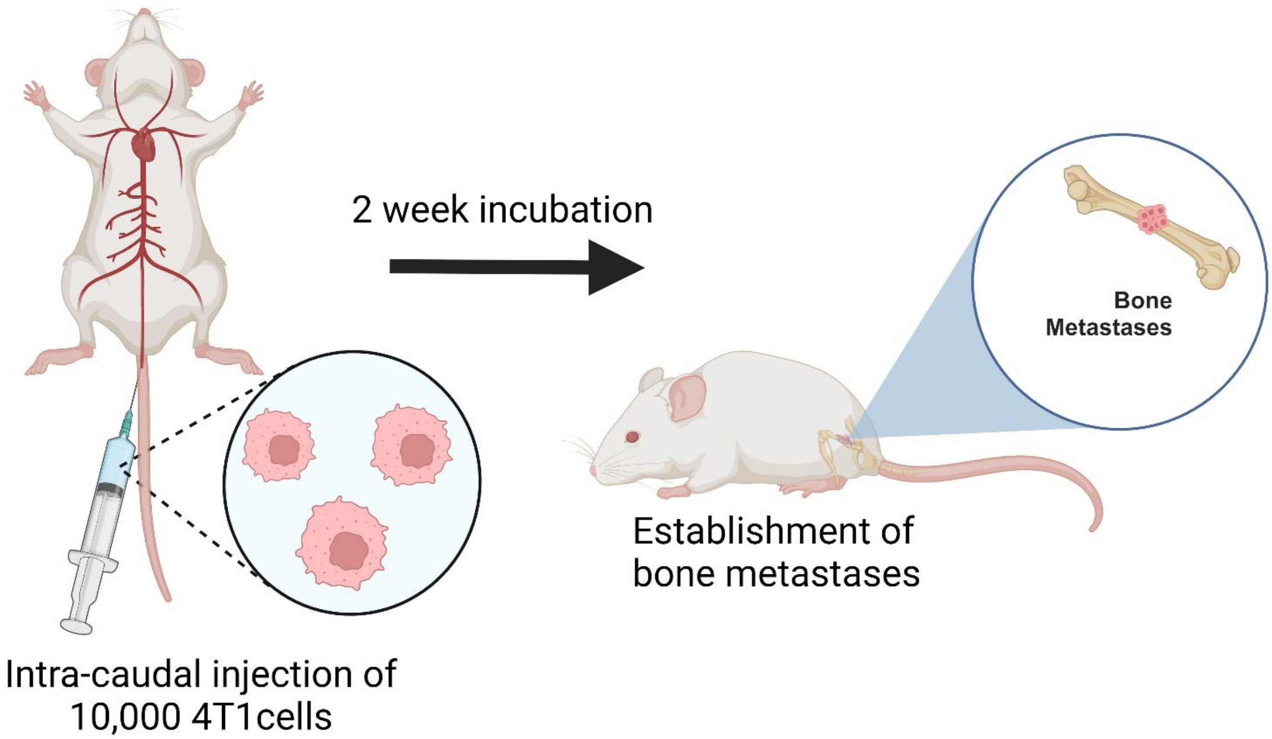

Figure S4: Scheme of intra-caudal injection of 4T1 cells to develop an in vivo bone metastases model.
